# Different Effects of Phototherapy for Rat Glioma during Sleep and Wakefulness

**DOI:** 10.3390/biomedicines12020262

**Published:** 2024-01-24

**Authors:** Alexander Shirokov, Inna Blokhina, Ivan Fedosov, Egor Ilyukov, Andrey Terskov, Dmitry Myagkov, Dmitry Tuktarov, Maria Tzoy, Viktoria Adushkina, Daria Zlatogosrkaya, Arina Evsyukova, Valeria Telnova, Alexander Dubrovsky, Alexander Dmitrenko, Maria Manzhaeva, Valeria Krupnova, Matvey Tuzhilkin, Inna Elezarova, Nikita Navolokin, Elena Saranceva, Tatyana Iskra, Ekaterina Lykova, Oxana Semyachkina-Glushkovskaya

**Affiliations:** 1 Institute of Biochemistry and Physiology of Plants and Microorganisms, Saratov Scientific Centre of the Russian Academy of Sciences, Prospekt Entuziastov 13, 410049 Saratov, Russia; 2 Department of Biology, Saratov State University, Astrakhanskaya Str. 83, 410012 Saratov, Russia; inna-474@yandex.ru (I.B.); terskow.andrey@gmail.com (A.T.); adushkina.info@mail.ru (V.A.); eloveda@mail.ru (D.Z.); arina-evsyukova@mail.ru (A.E.); ler.vinnick2012@yandex.ru (V.T.); admitrenko2001@mail.ru (A.D.); mariamang1412@gmail.com (M.M.); krupnova_0110@mail.ru (V.K.); tuzhilkinma@yandex.ru (M.T.); inna.elizarowa7@yandex.ru (I.E.); nik-navolokin@yandex.ru (N.N.); sophora68@mail.ru (E.S.); tata-isk@yandex.ru (T.I.); eckaterina_lykova@mail.ru (E.L.); 3 Physics Department, Saratov State University, Astrakhanskaya Str. 83, 410012 Saratov, Russia; fedosov_optics@mail.ru (I.F.); egor.re01@mail.ru (E.I.); dmyagk0v@yandex.ru (D.M.); ivanov.ivao@yandex.ru (D.T.); dethaos@bk.ru (M.T.); paskalkamal@mail.ru (A.D.); 4 Department of Pathological Anatomy, Saratov Medical State University, Bolshaya Kazachaya Str. 112, 410012 Saratov, Russia; 5 Physics Department, Humboldt University, Newtonstrasse 15, 12489 Berlin, Germany

**Keywords:** glioma, photobiomodulation, brain drainage, CD8+ cells, immune response

## Abstract

There is an association between sleep quality and glioma-specific outcomes, including survival. The critical role of sleep in survival among subjects with glioma may be due to sleep-induced activation of brain drainage (BD), that is dramatically suppressed in subjects with glioma. Emerging evidence demonstrates that photobiomodulation (PBM) is an effective technology for both the stimulation of BD and as an add-on therapy for glioma. Emerging evidence suggests that PBM during sleep stimulates BD more strongly than when awake. In this study on male *Wistar* rats, we clearly demonstrate that the PBM course during sleep vs. when awake more effectively suppresses glioma growth and increases survival compared with the control. The study of the mechanisms of this phenomenon revealed stronger effects of the PBM course in sleeping vs. awake rats on the stimulation of BD and an immune response against glioma, including an increase in the number of CD8+ in glioma cells, activation of apoptosis, and blockage of the proliferation of glioma cells. Our new technology for sleep-phototherapy opens a new strategy to improve the quality of medical care for patients with brain cancer, using promising smart-sleep and non-invasive approaches of glioma treatment.

## 1. Introduction

Sleep deficit is a common symptom of glioma, with growing evidence suggesting an association between sleep disturbance and poor physical and psychological outcomes, including survival [1,2,3,4,5,6]. One study using the General Sleep Disturbance Scale reported insomnia (disruption in sleep quantity, pattern, or architecture) in 100% of patients with glioma [7]. Other studies estimated the prevalence of sleep injuries in patients with glioma to be between 37.0 and 81.8% [8,9,10]. It is unknown whether sleep deficiency can be reliably correlated with glioma growth, but there are growing numbers of case reports to suggest that a connection is possible [6,11,12,13,14]. Indeed, Sadighi et al. found that specific sleep disturbances are contingent on the tumor’s location [12]. Glioma location is also an important factor in choosing a treatment plan. The sleep centers can be affected by glioma directly or by neuroinflammation and peritumoral edema from a growing tumor [11,13]. Subramanian et al. proposed that specific sleep disturbance induced by glioma growth could be a systemic marker of the initial location of glioma for surgical resection and be used as a potential tool to track recurrence [13]. The correlation between sleep disturbance, location of brain tumors, and survival is discussed in several excellent reviews [5,6,11,15]. However, its merits in the treatment of glioma and its recurrence have yet to be explored.

A decrease in resistance to glioma progression during the development of sleep deficiency may be associated with the suppression of brain drainage (BD), the activity of which is strictly dependent on sleep [16,17,18,19,20,21,22]. The reduced BD in subjects with glioma could be a reason for the accumulation of excess fluid in the skull, leading to a dramatic increase in the intracranial pressure [23]. Indeed, peritumoral edema promotes the accumulation of extensive brain fluids that is associated with high mortality in patients with glioma [23]. The reasons for the development of cerebral edema in glioma remain unknown. There is emerging evidence that reduced BD can play a crucial role in glioma progression [16,17,18]. Indeed, Ma et al. revealed that outflow of the cerebrospinal fluid (CSF) is reduced in glioma due to a blockage of circulation of the cerebral spinal fluid (CSF) [16]. The extensive accumulation of brain fluids aggravates the brain tumor microenvironment and attenuates intracranial drug delivery efficacy. The development of methods for the compensation of CSF outflow and restoring normal CSF circulation are worthy of clinical attention.

Recently, transcranial photobiomodulation (PBM) has been shown to effectively stimulate BD and the lymphatic removal of toxins from the brain [21,22,24,25,26,27,28]. There is evidence that the stimulating effects of PBM on BD are achieved by PBM-mediated regulation of the contraction and relaxation phases of the different lymphatic vessels (LVs) that we analyzed and discussed in our previous studies [26,27,28]. Indeed, using in vivo monitoring of cervical LVs carrying lymph from the brain to the neck lymph nodes or in the mesenteric lymphangion, we clearly showed PBM-induced stimulation of lymphatic contractility [27,28]. Furthermore, in in vitro experiments, we demonstrated that PBM induces a transient increase in the NO level in the culture of lymphatic endothelial cells obtained from the mesenteric lymphatics [28]. In ex vivo experiments, we found the PBM effects on the basal MLVs are suppressed by the blockage of NO generation [28]. Thus, PBM has both effects on the different LVs: stimulation of their constriction and an increase in NO production. The lymphatics produce NO during contraction as flow shear activates the endothelial cells [29,30,31]. The elevation of NO then contributes to the subsequent relaxation of different types of peripheral LVs. Traditionally, it was believed that NO suppressed lymphatic constriction. However, the NO-mediated regulation of lymphatics is not as simple as was first assumed. So, the basal NO in the mesenteric lymphatics increases with the frequency of contraction induced by systemic administration of saline [29]. In effect, elevated lymphatic pumping increases the NO production due to increased flow shear forces. In this case, NO provides a generalized inhibition of pumping during periods of high lymph flow. Thus, NO supports the relaxation of lymphatics after its constriction. The NO effects are different along LVs. The high NO production during lymphatic constriction is observed predominantly in the valves [29]. Indeed, the mesenteric lymphatic valves contain a 30–50% higher NO concentration than tubular regions during contraction due both to there being many endothelial cells and an increased expression of endothelial nitric oxide synthase. The NO generation in the lymphatic valves limits the pumped flow of the total lymphatics by lowering the frequency and stroke volume of individual contractions. Thus, despite the fact that all lymphatic endothelial cells are capable of generating NO in flow shear events during contractions [29], this does not mean that the role of NO is uniformly important for all sections of LVs during the contraction and relaxation process. From a physiological point of view, NO generated in the lymphatic valves during constriction diffuses into the flowing lymph, survives to downstream tubular sites, and contributes to tubular relaxation that is important for the peristaltic character of pumping [30,31].

Strikingly, PBM also suppresses glioma growth [24]. There is pioneering data demonstrating that PBM increases the resistance of rats to glioma growth and the survival of animals due to PBM-induced activation of apoptosis of glioma cells and a reduction in intracranial pressure through stimulation of BD [24]. Therefore, PBM may be a promising therapeutic approach in non-invasive treatment of glioma via photo-activation of the MLV functions and BD.

Taking into account all of the above, we have focused on solving the problem of the improvement of BD in Wistar rats with glioma using an innovative approach of PBM for MLVs under EEG control of sleep and wakefulness. The steroids are standard therapy for peritumoral edema surrounding gliomas [32]. However, steroid therapy has limited efficacy and significant side effects [33]. New therapeutic strategies targeting BD and the MLV functions appear to be promising [16,17,18]. The re-discovery of MLVs prompted a re-evaluation of the mechanisms responsible for regulation of BD and the formation of cerebral edema in gliomas [16,17,18,34]. In our series of experiments, for the first time, we clearly demonstrated that PBM stimulates BD in both healthy rodents and rats with glioma [24,25,26,27,28]. By studying the mechanisms of the therapeutic effects of PBM in the in vivo, ex vivo, and in vitro experiments, we discovered that PBM increases the contractility of LVs and increases their drainage properties [26,27,28]. Quite recently, in our study on mice with Alzheimer’s disease, we discovered that the effects of PBM on BD are stronger in sleeping vs. awake animals [21,22]. This phenomenon can be explained by the fact that during deep sleep, BD is activated due to an increase in the size of the perivascular spaces, which promotes the removal of toxins and metabolites dissolved in CSF [19]. Since in our previous work using PBM in awake rats we proved the suppression of glioma growth, we hypothesized that sleep could enhance the therapeutic effects of PBM due to better activation of BD. To test this hypothesis, in this study we compared the PBM effects during sleep and wakefulness on the glioma growth, including the apoptosis and proliferation of tumor cells, as well as on survival rate and BD, in Wistar male rats.

## 2. Materials and Methods

### 2.1. Subjects

Pathogen-free male *Wistar* rats (200–250 g, 2 months old) were used in all experiments and were obtained from the National Laboratory Animal Resource Centre (Shemyakin-Ovchinnikov Institute of Bioorganic Chemistry, RAS, Pushchino, Russia). The animals were housed under standard laboratory conditions with access to food and water ad libitum. All experimental procedures were performed in accordance with the “Guide for the Care and Use of Laboratory Animals”, Directive 2010/63/EU on the Protection of Animals Used for Scientific Purposes, and the guidelines from the Ministry of Science and High Education of the Russian Federation (№ 742 from 13 November 1984), which have been approved by the Bioethics Commission of the Saratov State University (Protocol No. 8, 18 April 2023). The mice were housed at 25 ± 2 °C, 55% humidity, and subject to a 12:12 h light–dark cycle (light: 08:00 a.m.–08:00 p.m.). The mice adapted to the experimental conditions during one week before the beginning of the experiments to ensure acclimation to the housing room of the animal facility. The experiments were performed in the following groups: (1) control (healthy rats) without the PBM course; (2) sham rats without the PBM course; (3) sham rats receiving the PBM course under EEG control of wakefulness; (4) sham rats receiving the PBM course under EEG control of deep sleep; (5) rats with glioma without the PBM course; (6) rats with glioma receiving the PBM course under EEG control of wakefulness; (7) rats with glioma receiving the PBM course under EEG control of deep sleep; *n* = 10 in each group in all sessions of the experiments and *n* = 20 in the study of survival rate.

### 2.2. Model of Rat Glioma

The C6 rat glioma cell line was obtained from the Russian Cell Culture Collection of Vertebrates, Institute of Cytology, Russian Academy of Sciences (St. Petersburg, Russia). A transfected C6—TurboRFP cell line was used for the study of the growth of fluorescent GBM [24]. The C6 cells were cultured in a Dulbecco’s Modified Eagle Medium (DMEM) growth medium (Paneco, Moscow, Russia) containing 2.5% embryonic veal serum (Biosera, Cholet, France), 4 mM glutamine (Paneco, Moscow, Russia), penicillin (50 IU/mL), and streptomycin (50 mg/mL) (Paneco, Moscow, Russia). Rat C6 glioma cells were transfected with TurboRFP-C DNA plasmids using the method of liposomal transfection followed by selection using geneticin (G418 antibiotic, neomycin analogue). The resulting cell line, C6—TurboRFP, has stable cultural and morphological characteristics.

The glioma cells (5 × 10^5^ cells per rat) were injected at coordinates AP—1 mm, ML—1 mm, DV—4 mm, with a Hamilton microsyringe in a volume of 15 µL at a rate of 1 µL/min. Physiological saline (15 µL, Sigma-Aldrich, St. Louis, MO, USA) was injected in the same region of the brain in the sham groups. Thereafter, the burr hole was sealed with sterile bone wax and tissue glue and the wound was sutured closed with 3–0 absorbable suture material. After the implantation of glioma cells, the wound was closed and treated with 2% brilliant green solution. The rats were removed from the stereotaxic head holder, given 0.01 mg/kg buprenorphine, s.c., and 50 K bicillin, i.m., returned to a temperature-controlled recovery cage, and moved back to the animal facility after recovery. Glioma growth and tumor volume were monitored using magnetic resonance imagining (MRI) 28 days after the tumor cell implantation using a Clin scan 7T tomograph (Bruker, Mannheim, Germany). The growth of fluorescent glioma C6—TurboRFP in the sham group and in rats that received the PBM course during wakefulness or sleep was assessed using confocal microscopy using a Nikon A1R MP confocal laser scanning microscope (Nikon Corp., Tokyo, Japan). For this purpose, 4 weeks after implantation of glioma cells, the brains of rats from the tested groups, including the control without PBM and the experimental groups that received PBM while asleep or awake, were removed and a whole fresh set of brains was scanned.

### 2.3. PBM under EEG Control

In this study, we used our adapted and previously published protocol for PBM under EEG control [35]. A two-channel cortical EEG was recorded. The rats were implanted with two silver electrodes (tip diameter: 2–3 µm) located at a depth of 150 µm at coordinates ML: 3.0 mm and AP: 3.0 mm from the bregma on either side of the midline under inhalation anesthesia with 1% isoflurane (Sigma-Aldrich, St. Louis, MO, USA), at a rate of 1 L/min (N_2_O/O_2_—70/30 ratio). The head plate was mounted and small burr holes were drilled. Afterward, EEG wire leads were inserted into the burr holes on one side of the midline between the skull and the underlying dura. The EEG leads were secured with dental acrylic (Zhermack SpA, Badia Polesine, Venice, Italy). Ibuprofen (Bhavishya Pharmaceuticals Pvt. Ltd., Hyderabad, Telangana, India; 15 mg/kg), for the relief of postoperative pain, was provided in the rats’ water supply for 2 to 3 days prior to surgery and for 3 days post-surgery. The rats were allowed 10 days to recover from surgery prior to beginning the experiment.

**Figure 1:**
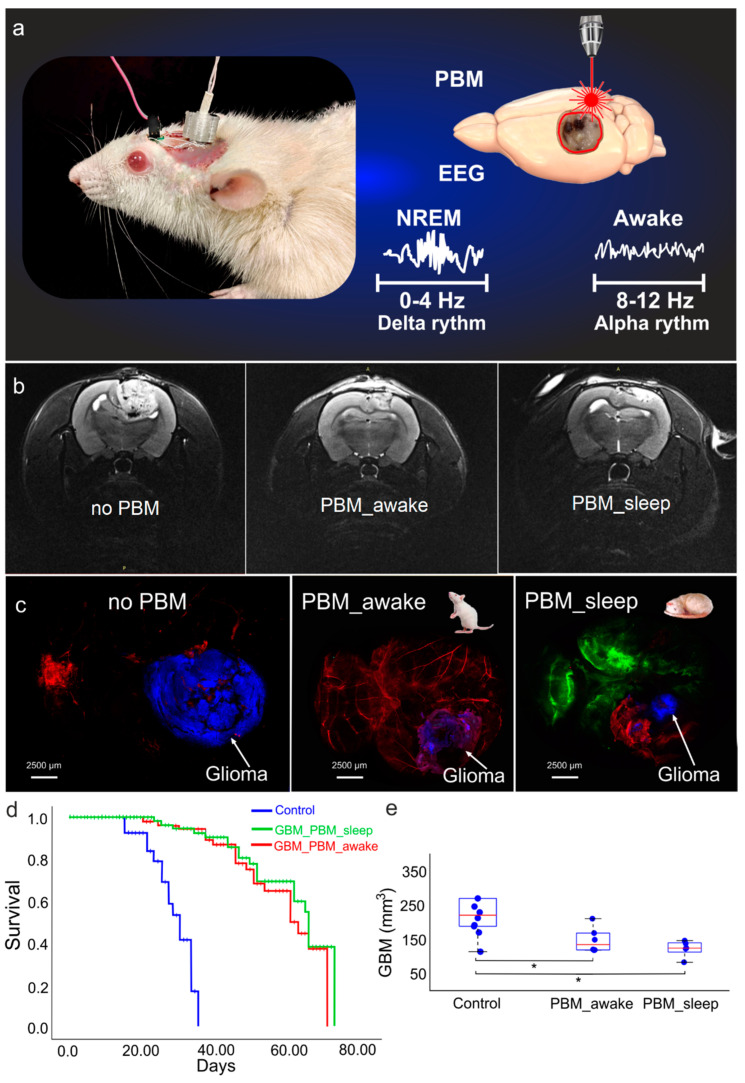
The effects of PBM on survival and progression of rats’ glioma: (**a**) schematic illustration of PBM under EEG control during sleep or awake states; (**b**) representative images of ex vivo confocal analysis of glioma size in rats without and after the PBM course during wakefulness or sleep, respectively; (**c**) representative MRI images of rat glioma 4–week growth without and after the PBM course during wakefulness or sleep; (**d**) Kaplan–Meier overall survival plots between the tested groups; the survival differences between rats with glioma, without, and after the course of PBM during sleep or wakefulness was significant (X2 test, *p* = 0.00000000000129 between the no PBM and the PBM_sleep groups and *p* = 0.00000000000024 between the no PBM and the PBM_awake groups; *n* = 20 in each group); (**e**) MRI analysis of glioma size in rats without and after the course of PBM during sleep or wakefulness; *n* = 10 in each group; *—*p* < 0.05 between groups, the ANOVA test with post hoc Duncan test.

EEG recording was performed with a tethered EEG system using an ADS1293 (Texas instruments, Dallas, TX, USA) low-power, 3-channel, 24-bit analog front-end for biopotential measurements. Initialization and data transfer were performed with an Atmega328 (Atmel, San Jose, CA, USA) microcontroller via an SPI interface. The same microcontroller was used to detect non-rapid eye movement (NREM) sleep in real time. The instrument was powered with an 18650 Li-ion battery. An ESP-01 (Espressif Systems, PRC) module was used for Wi-Fi communication between the instrument and standalone PC. The instrument was placed on top of the home cage and connected to the mouse with a 0.3 m long flexible 4-wire cable attached to a head-mounted miniature connector soldered to 4 screw electrodes. The connector allowed for the EEG instrument to be easily plugged into the mouse without anesthesia (Figure 1a). Note that anesthesia strongly affects the brain’s functions and BD, which makes it necessary to avoid the use of anesthesia in our experiments [36].

A 1050 nm LED with an output power of 50 mW in 2853 SMD housing was used for PBM. The LED driver was controlled with the pulse-width modulation (PWM) output of the EEG instrument microcontroller. The LED was connected to the instrument with a 0.3 m long 2-wire flexible cable and mounted into a miniature 3D printed frame with a pair of cylindrical magnets 3 mm in diameter and 3 mm in height each. That allowed for easy attachment of the LED frame to an M3 steel washer glued at the surface of the mouse’s skull. The LED operated in PWM mode at a 1 kHz modulation frequency. The washer hole, of 3.5 mm in diameter, acted as an aperture limiting the PBM area to 0.1 cm^2^ at the skull’s surface.

The LED output power of optical radiation was 50 mW and the area limited with a washer was 0.1 cm^2^. Thus, in continuous operation mode the power density was 500 mW/cm^2^, complying with ANSI Z136 for maximal permissible exposure (MPE) of skin to 1050 nm radiation. When operating in the pulse-width modulation (PWM) regime at 1 kHz with a duty cycle less than 10%, the instant and averaged power densities were both at least 10 times less than the MPE. A duty cycle of 2% corresponds to 10 mW/cm^2^. For a 17 min (1020 s) long procedure at 2% PWM duty, the LED delivers a 10 J/cm^2^ dose over the irradiated skull’s surface. The instrument was controlled with software developed using LabVIEW (NXG 5.1, National instruments, Dallas, TX, USA).

The software enables EEG recording, monitoring of the instrument’s operation, and remote configuration of the NREM sleep detection and PBM procedure. The EEG signal was digitized at 2.4 kSa/s, and filtered with a fifth-order digital Butterworth bandpass filter with a lower cutoff frequency of 0.5 Hz and an upper one of 250 Hz. A detailed description of the method is given in our article [35]. When 20% of the 30 s epochs (6 of 30 consecutive 1 s records) were scored as NREM [37], this was defined as the NREM sleep stage of the animal, and the PBM LED was automatically turned on to run for 17 min at the given PBM. Once configured, the instrument was operated autonomously, logging its operations to be monitored via a Wi-Fi connection. Wakefulness, NREM, and rapid eye movement (REM) sleep were defined as described in our previous studies, where we demonstrate the EEG patterns and spectrum characteristics of wakefulness, NREM, and REM sleep in rodents [21,22].

### 2.4. Optical Monitoring of Brain’s Drainage System

To study the PBM effects on BD, we analyzed spreading of fluorescein isothiocyanate (FITC)-dextran (FITCD, Sigma, St. Louis, MO, USA) in the dorsal and ventral parts of the whole brain after a single PBM application using ex vivo confocal microscopy. An amount of 7 μL of FITCD was injected into the right lateral ventricle (AP—1.0 mm; ML—1.4 mm; DV—3.5 mm) at a rate of 0.1 μL/min using a microinjector (Stoelting, St. Luis, USA) with a Hamilton syringe with a 29-G needle (Hamilton Bonaduz AG, Switzerland). The implantation of a chronical polyethylene catheter (PE-10, 0.28 mm ID × 0.61 mm OD, Scientific Commodities Inc., Lake Havasu City, AZ, USA) into the right lateral ventricle was preformed according to the protocol reported by Devos et al. [38].

The head plate with LED was fixed in the region of the parietal and interparietal bones using dental acrylic (Zhermack SpA, Badia Polesine, Italia) under inhalation anesthesia with 1% isoflurane (Sigma-Aldrich, St. Louis, MO, USA), at a rate of 1 L/min (N_2_O/O_2_—70/30 ratio). The LED was fixed to the head plate with two screws and was placed on the parietal region, where the largest number of MLVs is located [39] (Figure 1a). The LED was performed for 61 min using the following sequence, 17 min—LED, 5 min—pause, 17 min—LED, 5 min—pause, 17 min—LED, as we have determined by a random selection in our previous studies [21,22,24,35]. The irradiance at the skull’s surface did not exceed 0.5 W/cm^2^. The dose for PBM during a single PBM treatment was 30 J/cm^2^ and for the 14 days of the PBM course it was 0.42 kJ/cm^2^. Using this PBM dose, we did not find any changes in temperature on the brain surface after PBM, consistent with the results of our previous study [24].

A type A-K3 thermocouple (Ellab, Hillerød, Denmark) was used to measure the skull’s temperature. The thermocouple was placed subcutaneously 2 mm lateral to the bregma in the irradiated zone. A burr hole was drilled under inhalation anesthesia (1% isoflurane at 1 L/min; N_2_O/O_2_—70:30). To measure the brain surface temperature under 1050 nm LED irradiation, the medial part of the left temporal muscle was detached from the skull bone, a small burr hole was drilled into the temporal bone, and a flexible thermocouple probe (IT-23, 0.23 mm diameter, Physitemp Instruments LLC, Clifton, NJ, USA) was introduced between the parietal bone and the brain into the epidural space. Brain surface temperature was measured before and during laser stimulation with a 5 min increment using a handheld thermometer (BAT-7001H, Physitemp Instruments LLC, Clifton, NJ, USA).

To study the PBM effects on BD during wakefulness and deep sleep, the intracerebroventricular injection of FITCD was performed at 08:00 a.m. under the EEG monitoring when awake and after 08:00 p.m. at the time of EEG monitoring of NREM for a 3 h observation period. The times of 8 a.m. and 8 p.m. for the intracerebroventricular injection of FITCD were chosen due to the light regime of the vivarium and to standardize the protocol to start the experiment at the time of the natural transition to sleep or wakefulness. To keep the same time for the distribution of FITCD in the awake and sleeping states, rats that did not show NREM during the 3 h observation period were not included in the studies. The ex vivo optical study of the FITCD distribution in the brain fluid system was performed 3 h after the intracerebroventricular injection of FITCD. Afterward, whole-brain imaging from the dorsal and ventral aspects as well as the deep cervical lymph nodes (dcLNs) was performed using confocal microscopy (Nikon Corp., Tokyo, Japan).

The 14-day PBM course during sleep or wakefulness was performed daily in rats with 4-week-old glioma. The PBM course under ECG control was performed during observation of NREM (synchronized activity with high amplitude, which is dominated by low-frequency delta waves (0–4 Hz) comprising >30% of EEG waveforms/epochs) or awake (low-amplitude and high-frequency dynamics >10%, 8–12 Hz).

The imaging was performed using a Nikon A1R MP upright confocal microscope (Nikon Corp., Tokyo, Japan) with CFI Plan Apochromat Lambda D 2X (Nikon Corp., Tokyo, Japan) installed in a focusing nosepiece. The brain samples were submerged in a buffer solution in a Petri dish placed on the microscope stage. The top surface of each sample was covered with a 25 mm × 25 mm × 0.17 mm glass cover slide. The brain image was captured as a stack of 5 stitched large images each over 6 × 6 fields of view; the dcLNs image was captured as a stack of 4 stitched large images each over 2 × 2 fields of view. The image resolution of the brain was 3636 × 3636 pixels at 6.11 µm/pixel and for dcLN 0.3218 µm/pixel. The Z step was 222 µm. The resulting image was obtained as a maximum-intensity Z projection of all 5 images of the stack. The method enables a high-resolution image to be obtained extended along a 1 mm depth of focus. The confocal images were captured in two channels: 488 nm excitation/500–550 emission was used to image the FITCD distribution; and 640 nm excitation/663–738 nm emission for Evans Blue dye (1%, Sigma-Aldrich, St. Louis, MO, USA) that was intravenously injected 30 min before the experiments for labeling of the cerebral blood vessels. The dcLNs were imaged identically but only a single field of view was captured for each stack. Image processing was performed using the Fiji open-source image-processing package [40]. The image-processing procedures were identical for each pair of images (control and laser-treated samples) for each channel to ensure an accurate comparison of the fluorescence intensity.

### 2.5. MRI of Rat Glioma

A tumor-volume assessment was performed in the 4th week after GBM implantation using a 7 Tesla Bruker BioSpec 70/30 USR dedicated research MRI scanner and the Paravision 6.0 data acquisition software (Bruker Biospin; Billerica, MA, USA). To obtain a good signal-to-noise ratio, a 72 mm small-bore linear RF volume coil with an actively decoupled brain surface coil (40 cm bore; 660 mT/m, rise time within 120 μs) was used for excitation and signal detection. Rats were anesthetized with 1% isoflurane at 1 L/min N_2_O/O_2_ (70:30). Temperature and respiration were monitored and maintained using a thermal air blower. Anatomical T2-weighted images were acquired with a fast spin echo sequence (rapid acquisition with relaxation enhancement; repetition time (TR)/echo time =  5000 ms/56 ms; field of view =  4 cm × 4 cm; slice thickness  =  1 mm; slice gap (inter-slice distance)  =  1.1 mm; number of slices  =  12; matrix  =  256 × 256; number of averaging  =  3) as previously described [41]. T1-weighted imaging used the RARE technique, with 9.6 ms TE, 1000 ms TR, and a RARE factor of 2, thus 4 averages, requiring 4 min 16 s to assess tumor volumes; ROIs were drawn around regions of visible hyperenhancement in each of the slices on the T2-weighted and corresponding T1-weighted images using NIH ImageJ and calculated using the MATLAB software (version 2018b, MathWorks, Inc., Natick, MA, USA). Statistical analysis was performed using GraphPad Prizm v.6.0.

### 2.6. Immunohistochemistry (IHC)

Rats were euthanized with an intraperitoneal injection of a lethal dose of ketamine and xylazine and intracardially perfused with 0.1 M of PBS for 5 min. Afterward, their brains were removed and fixed in 10% buffered formalin with wiring material in alcohol, and poured into paraffin. The paraffin sections were stained with hematoxylin and eosin, and IHC studies were performed using the REVEAL Polyvalent HRP-DAB detection system. The monoclonal antibodies (Abcam, Cambridge, UK) Bcl (MAB8272), Ki67 (clone SP6, ab16667), and p53 (ab131442) were used at a dilution of 1:100 to the antibody. When staining with ICH markers, positive and negative controls were used to exclude false-negative and false-positive results, to create standardization of the staining conditions and increase the objectivity of the results. The percentage of positively expressing cells in 10 fields of view for each sample and the intensity of the immunohistochemical reactions were calculated. All studies were performed using a MicroVisor of medical transmitted light µVizo-103 (LOMO, St. Petersburg, Russia) with a magnification of 640 times.

For confocal imaging of the brain slices and the dcLNs we used the protocol for the IHC analysis with the markers for the transmembrane glycoprotein surface marker CD8+ T lymphocytes (CD8) for lymphatic vessel endothelial hyaluronan receptor 1 (LYVE1).

For the IHC analysis, brain and dcLN tissues were collected and free-floating sections were prepared. Tissue of the brain and dcLN were fixed for 48 h in a 4% saline-solution-buffered formalin, then sections of the brain and dcLN with a thickness of 40–50 microns were cut on a vibrotome (Leica, Wetzlar, Germany). The tissues of dcLN were previously poured into 2% agarose on saline solution. The sections were processed according to the standard immunohistochemical protocol with the corresponding primary and secondary antibodies. The sections of mouse brain and dcLN were imaged using a Leica SP8 confocal laser scanning microscope (Leica, Wetzlar, Germany). The antigen expression was evaluated on free-floating sections according to the standard method of simultaneous combined staining (Abcam protocols for free-floating sections).

The nonspecific activity was blocked by 2 h of incubation at room temperature with 10% BSA in a solution of 0.2% Triton X-100 in PBS. Solubilization of cell membranes was carried out during 1 h of incubation at room temperature in a solution of 1% Triton X-100 in PBS. The sections of the brain and of the dcLNs were, firstly, washed 3 times (5 min for each) with wash solution (0.2% Triton X-100 in PBS), secondly incubated in the blocking solution (a mixture of 0.2% Triton X-100 and 10% BSA in PBS) for 2 h, followed by incubation with rat anti-CD8+ antibody (1:500; ab 22378, Abcam, Cambridge, UK) and rabbit anti-Lyve-1 antibody (1:500; ab 218535, Abcam, Cambridge, UK) overnight at 4 °C in PBS containing 0.2% Triton X-100 and 0.5% normal goat serum. Next, samples were incubated at room temperature for 1 h and then washed 3 times, followed by incubation with goat anti-rat IgG (H+L) Alexa Fluor 488 (Invitrogen, Molecular Probes, Eugene, OR, USA), and goat anti-rabbit IgG (H+L) Alexa Fluor 555 (Invitrogen, Molecular Probes, Eugene, OR, USA) for visualizing the LVs in the dcLNs. In the final stage, the sections were transferred to the glass and 15 µL of mounting liquid (50% glycerin in PBS with DAPI at a concentration of 2 µg/mL) was applied to the section, then the preparation was covered with a cover glass and confocal microscopy was performed.

The sections of brains and dcLN were visualized using a confocal microscope (Nikon A1R MP, Nikon Instruments Inc., Melville, New York, USA) with a ×20 lens (0.75 NA), or a ×100 lens for immersion in oil (0.45 NA). DAPI, Alexa Fluor 488, and Alexa Fluor 555 were excited with excitation wavelengths of 405 nm, 488 nm, and 561 nm, respectively. Alexa Fluor 647 and Evans Blue were excited with the same excitation wavelength of 647 nm. Three-dimensional visualization data were collected by obtaining images in the x, y, and z planes. The images were obtained using the NIS-Elements software (v.5.21, Nikon Instruments Inc.) and analyzed using Fiji software (v.2.9.0, open-source image processing software) and Vaa3D (v. 1.1.4, open-source visualization and analysis software).

### 2.7. Statistical Analysis

All statistical analyses were performed using the Microsoft Office Excel and SPSS 17.0 for Windows software. The results were reported as a mean value ± standard error of the mean (SEM). The inter-group differences in all series experiments were evaluated using the ANOVA test with post hoc Duncan test. The significance levels were set at *p* < 0.05 for all analyses. No statistical methods were used to predetermine the sample size.

## 3. Results

### 3.1. The Effects of the PBM Course during Sleep or Awake State on Survival and Glioma Progression

In our previous study, we clearly showed that the therapeutic effects of PBM are higher in sleeping vs. awake animals with Alzheimer’s disease [21,22]. Since in our recent investigation on awake rats we discovered that PBM might also be a promising tool for the suppression of glioma growth, here we compare the therapeutic effects of PBM during the sleep and awake states.

In the first step, we studied the effect of the PBM course on the survival rate and the tumor volume in sleeping and awake rats. The MRI data clearly show that the glioma volume was significantly decreased in both the PBM groups vs. the no PBM group (Figure 1b,e). However, the glioma volume reduction was 1.3-fold greater in rats receiving PBM during sleep than during wakefulness (Figure 1b,e).

Additionally, we performed confocal analysis of the glioma volume in the tested groups using our original model of fluorescent glioma (Figure 1c). The ex vivo confocal data also demonstrate that the effects of PBM on the suppression of glioma growth was significantly higher in the PBM_sleep group vs. the PBM_awake group.

The survival rate was evaluated using the Kaplan–Meier method. Figure 1d clearly demonstrates that the survival rate was significantly higher in both the PBM groups than in rats with glioma without PBM (X2 test, *p* = 0.00000000000129 between the no PBM and the PBM_sleep groups and *p* = 0.00000000000024 between the no PBM and the PBM_awake groups; *n* = 20 in each group). There were no differences in survival between rats receiving PBM during the sleep and awake states.

This series of experiments shows that although the courses of PBM in sleeping and awake rats increased their survival equally well, the decrease in glioma volume was more pronounced in rats receiving PBM in the sleep than in the awake state.

### 3.2. The Effects of the PBM Course during Sleep or Awake State on Apoptosis and Proliferation of Glioma Cells

**Figure 2:**
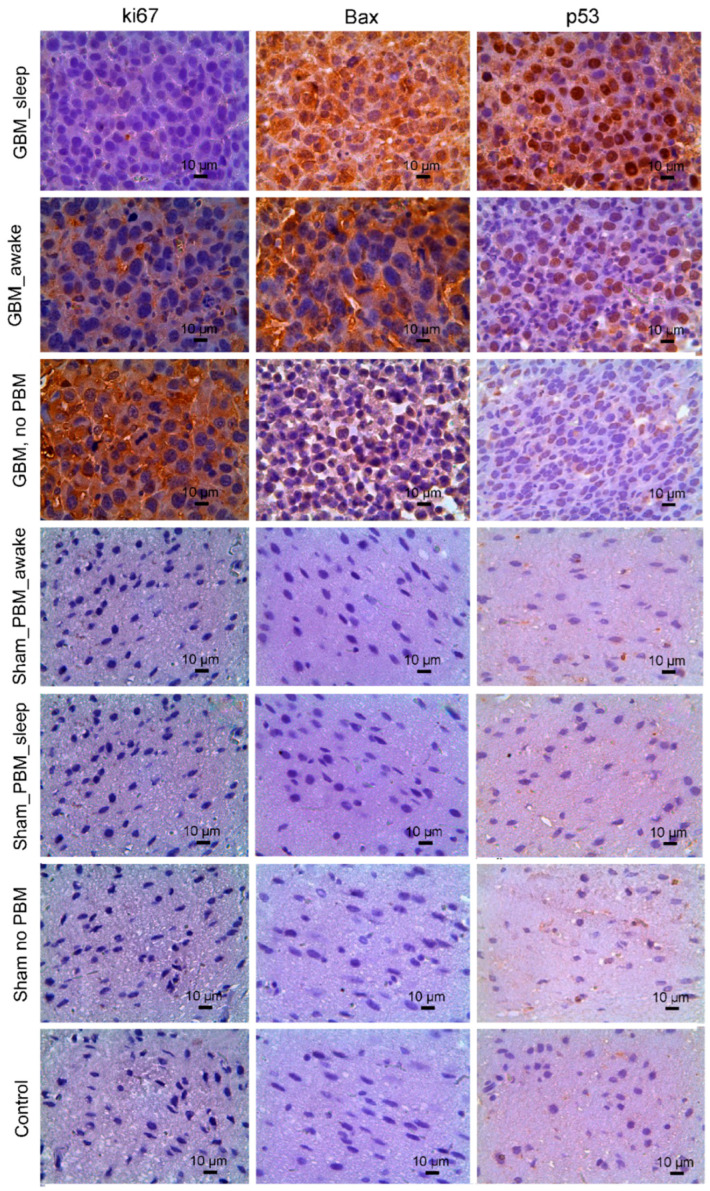
IHC analysis of proliferation and apoptosis of glioma cells in seven tested groups, including control (healthy rats) group; 3 sham groups, in which animals received an injection of saline in the same volume and location as the glioma cells were implanted without PBM and after the PBM course during sleep and awake states; 3 glioma groups, in which rats were with glioma without PBM and after the PBM course during sleep and awake states. The quantitative analysis of immunopositive cells (shown in brown) expressing K67, Bax, and p53 is presented in Table 1.

The activation of the apoptosis of glioma cells and the suppression of their proliferation underlie the suppressive effects of PBM on the glioma growth, as we established in our recent studies in awake rats [24]. Hypothesizing that PBM during sleep could have stronger effects on the glioma progression than during the awake state, in the second step, we studied the cellular mechanisms of the therapeutic effects of the PBM course in sleeping and awake rats with glioma (Table 1, Figure 2).

The data of the IHC study show that the expression of the marker of proliferation (Ki67) was higher in rats with glioma and without the PBM course vs. rats treated by the PBM course during the sleep or awake state. However, the proliferation of glioma cells was suppressed more strongly in rats that received the PBM course during the sleep vs. the awake state.

The expression of the marker of apoptosis Bax, with p53 as its regulating factor, was lower in glioma without the PBM course vs. the PBM course during the sleep and awake states (Table 1). Notably, activation of apoptosis in glioma cells was significantly higher in rats receiving the PBM course during the sleep compared to the awake state.

Thus, both PBM courses during sleep or wakefulness effectively increased resistance to the progression of glioma via suppression of proliferation of the tumor cells and activation of apoptosis. Interestingly, the effects of the PBM course on the expression of Ki67, Bax, and p53 were 2.3-, 1.5-, and 1.8-fold greater in rats receiving the PBM course during the sleep vs. the awake state (Table 1).

### 3.3. The Effects of the PBM Course during Sleep or Awake State on BD

Deep sleep is accompanied by an increase in the size of the perivascular spaces, which facilitates the movement of brain fluids and promotes activation of BD [19]. Since in our previous study on awake rats we found that PBM can stimulate BD, here we tested our hypothesis that BD will be more strongly activated by PBM in sleeping vs. awake rats due to a PBM-related enhancement in natural BD activation.

**Figure 3:**
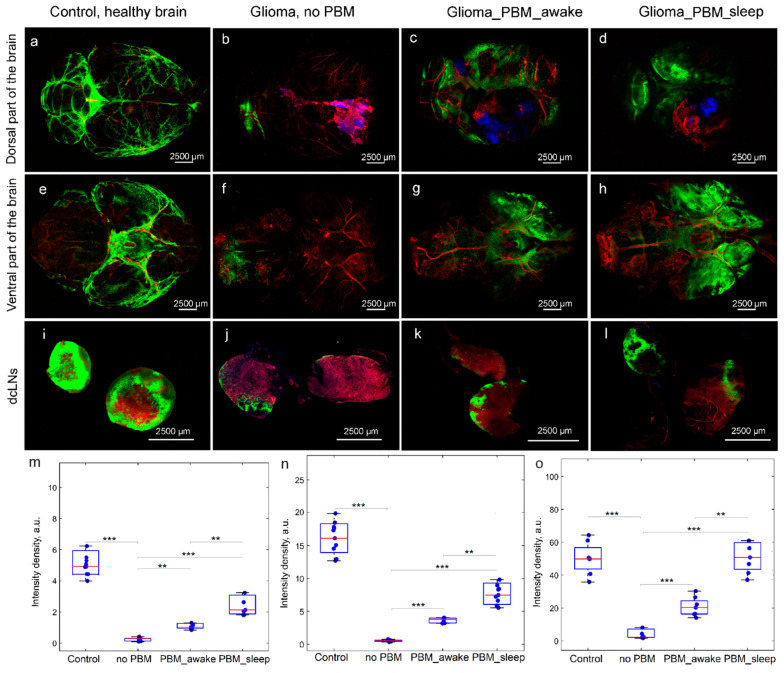
The effects of the PBM course during sleep or wakefulness on BD in the tested groups: (**a**–**d**) and (**e**–**h**) Representative images of FITCD spreading in dorsal (**a**–**d**) and ventral (**e**–**h**) parts of the brain from the control group (healthy brain) (**a**,**e**), the glioma group without the PBM course (**b**,**f**), and the groups treated by the PBM course during wakefulness (**c**,**g**) or sleep (**d**,**h**); (**i**–**l**) representative images of accumulation of FITCD in dcLNs in rats from the control group (**i**), the glioma group without the PBM course (**j**), and the groups treated by the PBM course during wakefulness (**k**) or sleep (**l**); (**m**–**o**) quantitative analysis of intensity of fluorescent signal from FITCD in dorsal (**m**) and ventral (**n**) parts of the brain and in dcLNs (**o**); *n* = 10 in each group; ***—*p* < 0.001; **—*p* < 0.01 between groups; the ANOVA test with post hoc Duncan test.

For this purpose, the lymphatic removal of FITCD from the brain to the dcLNs was studied. The results of ex vivo confocal imaging of the whole brain revealed a significant reduction in FITCD spreading in brain tissues and its lymphatic removal to the dcLNs in rats with glioma without the PBM course vs. the control, including healthy rats (Figure 3a,b,e,f,i,j).

Indeed, the quantitative analysis found that the intensity of the fluorescent signal from FITCD in the ventral and dorsal parts of the brain, as well as in the dcLNs, was 7.5-, 9.3-, and 5.1-fold greater in healthy rats vs. rats with glioma, respectively (Figure 3m–o). Both PBM courses during sleep or wakefulness essentially improved the FITCD distribution in brain tissues and its further accumulation in the dcLNs (Figure 3b–d,f–h,j–o). However, the stimulating PBM effects on BD was higher in rats receiving the PBM course during sleep vs. when awake (Figure 3c,d,g,h,k–o). Thus, these findings demonstrate that glioma-mediated suppression of BD can be improved by the PBM course, with more pronounced effects of PBM during the sleep vs. the awake state.

### 3.4. The Effects of the PBM Course during Sleep or Wakefulness on Brain’s Tumor Immunity

Accumulating evidence indicates that increasing cytotoxic CD8+ T cell infiltration improves survival in glioma [42]. Indeed, activated CD8+ T cells activate the apoptosis, providing suppression of tumor growth [43,44,45]. However, the lack of signaling molecules in glioma, which activate the generation of lymphocytes in the lymph nodes, is a main reason for the limitation of CD8+ T cells in the tumor region, leading to immune suppression [46,47]. Therefore, in the next step, we investigated the stimulating effects of the PBM course during the sleep or awake state on the immune response against glioma.

**Figure 4:**
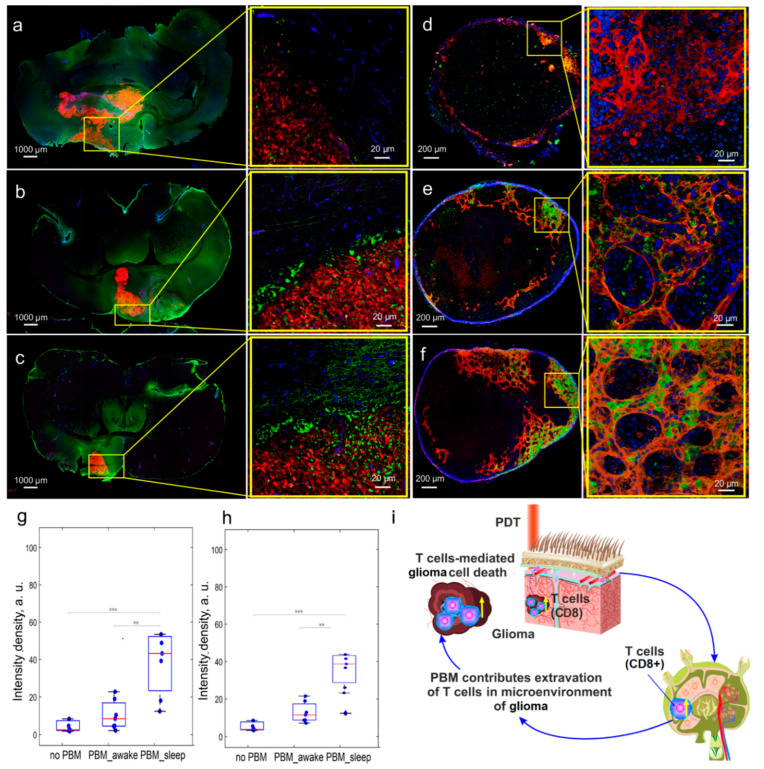
The effects of the PBM course during sleep or wakefulness on brain’s tumor immunity: (**a**–**f**) Representative images of fluorescent glioma (red) and CD8+ T cells (green) in the brain (**a**–**c**) and in dcLNs (**d**–**f**) in rats with glioma and without the PBM course (**a**,**d**), in rats with glioma receiving the PBM course during wakefulness (**b**,**e**), and in rats with glioma and receiving the PBM course during sleep (**c**,**f**); in (**d**–**f**): CD8+ T cells (green), LVs (red), and DAPI (blue); (**g**,**h**)—quantitative analysis of intensity of fluorescent signal from CD8+ T cells in glioma (**g**) and in dcLNs (**h**); *n* = 10 in each group, ***—*p* < 0.001; **—*p* < 0.01 between groups; the ANOVA test with post hoc Duncan test; (**i**)—schematic illustration of the PBM effects on an immune response against the glioma growth via increasing the number of cytotoxic CD8+ cells in glioma cells and in dcLNs.

Figure 4a,d,g,h demonstrate only a small number of CD8+ T cells around the fluorescent glioma and in the dcLNs in rats with glioma and without the PBM course. Notably, the number of CD8+ T cells in glioma cells and in the dcLNs was significantly increased after the PBM course, both during sleep or wakefulness (Figure 4a–h). However, the PBM course during sleep more strongly increases the number of CD8+ T cells in glioma and in the dcLNs than during awake state (Figure 4b,c,e–h). These data clearly show that the PBM course activates an immune response against the glioma growth that is more effective if PBM is applied during sleep than during wakefulness. Figure 4i schematically illustrates the PBM-mediated stimulation of the generation of CD8+ cells in dcLNs and their extravasation into the microenvironment of the glioma.

## 4. Discussion

In this study, we clearly demonstrate that PBM has a therapeutic effect on rat glioma that is consistent with our previous data [24]. Notably, the PBM course during sleep suppresses glioma progression more strongly and increases survival compared to PBM during the awake state. These results were expected since we assumed that PBM during sleep increases BD, as we also showed in our earlier studies [21,22]. Indeed, we found dramatically reduced BD in rats with glioma that was significantly improved after both the PBM courses during either the sleep or awake state. However, the PBM effects on BD were statistically higher in sleeping vs. awake rats. This can be explained by the fact that sleep is associated with the activation of BD and lymphatic removal of metabolites and toxins from the brain [19,20,21,22]. Thus, PBM during sleep affects naturally activated BD. The reduced BD in subjects with glioma is accompanied by a lack of MLVs [16,17,18]. Therefore, intracisternal injection of the vascular endothelial growth factor C for stimulation of lymphaneogenesis is proposed as a new promising strategy for the treatment of brain tumors [18]. PBM can also improve the MLV functions, as we clearly demonstrated in our previous study using photoablation of MLVs [22]. In this study, we show that PBM-mediated restoration of cleaning and drainage functions of MLVs after injuries is better after PBM during sleep vs. when awake [22]. Interestingly, the PBM course during sleep vs. when awake promotes better learning in mice [35].

The mechanisms of PBM-suppression of glioma and the improvement of survival could be due to PBM-mediated activation of apoptosis and blockage of the proliferation of glioma cells. These effects of PBM were also more pronounced in sleeping vs. awake rats. These data are confirmed by our previous findings of the cellular mechanisms of phototherapy for glioma [24]. The application of PBM during sleep-associated activation of BD can contribute to the optimization of immune responses against glioma due to improvements in the microenvironment of the tumor and compensation of the CSF outflow. Indeed, we revealed a lack of CD8+ T cells in both brain tumors and in dcLNs in rats with glioma that has also been shown in other studies [46,47]. However, PBM significantly improved the number of tumor-infiltrating cytotoxic CD8+ T cells more significantly in sleeping vs. awake rats. Nakano et al. report that an increased proliferative activity of CD8+ T cells associated with a decrease in the Ki67 expression is a favorable prognostic factor for survival in patients with cancer [48]. CD8+ T cells can produce antitumor antibodies, support antitumor immune responses, and help to prime immune responses at the tumor site [44,49].

Currently, photodynamic therapy (PDT) is considered as a promising tool as an alternative therapy for glioma [50,51,52,53,54,55]. PDT combines a light source, which excites a photosensitizer accumulated specifically in the glioma cells. The excited photosensitizer interacts with triplet oxygen and produces singlet oxygen, damaging the tumor cells by direct (necrosis and apoptosis) and indirect (occlusion of tumor vessels and enhanced host immunity) injuries [51]. There are several clinical studies suggesting the therapeutic effects of PDT in patients with glioma using different photosensitizers, as we discussed in our review [51]. However, PDT cannot be used in patients with allergies to photosensitizers, including newborns, due to side effects of the photosensitizers. Indeed, there are disadvantages of PDT, including photosensitivity after treatment, erythema, edema, blisters, urticarial, photophobia, heat-shock response, and an increase in the permeability of the blood–brain barrier [56,57,58,59,60]. Recently, we proposed a new strategy for phototherapy for glioma, using PBM without photosensitizers in awake animals [24]. There is emerging evidence that sleep can significantly increase the therapeutic properties of PBM [21,22,35]. However, the idea of PDT of brain diseases during sleep is in its infancy [61]. Therefore, there are no technologies for simultaneous PBM and sleep monitoring [61]. In this study, we propose the effective application of our new technology for sleep-phototherapy for glioma that incorporates modern state-of-the-art facilities of optoelectronics and biopotential detection and that can be built of relatively cheap and commercially available components.

## 5. Conclusions

In this study, using new technology for sleep-phototherapy for glioma we showed for the first time that the therapeutic effects of PBM during sleep in rats with glioma are significantly enhanced compared with the use of PBM during the awake state. Indeed, the PBM course in sleeping rats vs. awake ones suppresses glioma growth more strongly and increases survival compared with the control. A new result was the fact that the PBM course during the sleep vs. awake state has greater effects on the immune response against glioma, including an increase in the number of cytotoxic CD8+ in glioma cells that was associated with activation of apoptosis on the glioma cells and suppression of their proliferation. We also found stronger stimulation of BD by PBM in sleeping vs. awake rats, which is consistent with our findings in previous studies [21,22]. Our new technology for sleep-phototherapy for glioma opens a new strategy to improve the quality of medical care for patients with brain cancer using promising smart-sleep and non-invasive approaches of glioma treatment. 

## Figures and Tables

**Table 1:** Markers of proliferation and apoptosis (%) in the tested groups; *n* = 10 in each group; ***—*p* < 0.001; **—*p* < 0.01—between rat glioma without and after PBM; †††—*p* < 0.001; ††—*p* < 0.01 between PBM during sleep or wakefulness; *n* = 10 in each group; the ANOVA test with post hoc Duncan test.

Markers	Control (Healthy Rats)	Control (Sham Rats)	Control (Sham Rats PBM_Sleep)	Control (Sham Rats PBM_Awake)	Rats with Glioma, No PBM	Rats with Glioma, PBM_Awake	Rats with Glioma, PBM_Sleep
Ki67	-	-	-	-	98.87 ± 1.1	27.44 ± 6.0 ***	11.69 ± 1.0 ***†††
Bax	-	-	-	-	16.29 ± 4.3	65.33 ± 7.5 **	96.91 ± 3.8 ***††
p53	-	-	-	-	20.25 ± 6.1	47.00 ± 4.4 **	87.33 ± 7.4 ***†††

## Data Availability

The data that support the findings of this study are available on request from the corresponding author.
